# Transnational Nationalism and Idealistic Science: The Alcohol Question between the Wars

**DOI:** 10.1093/shm/hkv138

**Published:** 2016-04-06

**Authors:** Johan Edman

**Keywords:** alcohol, conferences, interwar years, prohibition, temperance, eugenics, scientification

## Abstract

This article studies the interwar international conferences on the alcohol problem. How did they view the alcohol problem and its causes; what were the consequences for the individual and the society as a whole; and which solutions merited discussion? The first post-war conferences enjoyed an optimistic and internationalistic atmosphere, added to by American prohibition, which had given the temperance movement plenty to be hopeful about. But when the 1920s turned to the 1930s, the conferences were transformed into arenas for national solutions and into outright propaganda pieces. The responses to the alcohol problem debated in the interwar conferences built on a combination of scientifically masked ideological conviction and ideologically inspired passion for science. The apparently neutral ethics of such thinking was manifested in various radical measures to combat alcohol abuse.

## Introduction

When the conference delegates of the 15th International Congress against Alcoholism came together in Washington, DC at the end of September 1920, the circumstances were in many ways very different from the setting of the last pre-war conference in Milan in 1913. The war had left a mark on the alcohol question, for the war-time mobilisation and the time of crisis had led to widespread alcohol restrictions. Russia had introduced prohibition as early as 1914 as part of the war effort, and would go on to maintain it until 1925.^1^ And in Washington, DC in 1920, the conference participants convened for the first time in a country that had introduced total prohibition, which had been aired and petitioned in these conferences ever since the late 1800s.

The special circumstances of the interwar years changed how the alcohol problem was formulated in many other ways. The First World War had shaken the trust in transnational exchange and collaboration which lay at the very core of the international conferences. This trauma also gave rise to the ideological antagonisms of the interwar period, with fascist and Nazi regimes in Italy, Germany and Spain, and a Bolshevik takeover in Russia. The interwar years made room for a considerably greater range of views at the conferences as to what the state, politics and civil society could achieve than had been the case in the early twentieth century.

This article studies the international conferences on the alcohol problem, arranged in 1920–1939 by the International Temperance Bureau (ITB, renamed in 1923 as the International Bureau Against Alcoholism, IBAA). While it may be somewhat misleading to treat the interwar years as a uniform entity, the conferences did reflect a concentration on certain topics all through the period. I will therefore focus on continuity rather than change, but will also comment on recognisable discontinuities during this period.

As historical and social science research has shown, alcohol and drug issues have functioned as a catalyst for more comprehensive questions such as youth unemployment, nationalism or anti-modernity. The aim of this article is both to characterise the general themes that were discussed in the name of the alcohol problem and also to investigate how the alcohol question was framed in terms of these themes.^2^ How did these societies depict the alcohol problem and its causes? What were the consequences for the individual and the society as a whole? Which solutions merited discussion? Was there any common ground in terms of, for instance, generally accepted scientific descriptions of the alcohol problem?

The source material comprises the conference proceedings of seven of the eight international alcohol conferences held during 1920–1939 (see Table 1).^3^ These proceedings tell us who the participants were and which organisations were present. The reports give us access to formal speeches and other manifestations of conference etiquette, to minuted discussions and—most important in terms of this study—to the participants’ papers which had been dispatched and were read aloud in the conference. These debated a range of issues, including the impact of alcohol on the brain, relevant restrictive systems, the significance of female citizenship on the alcohol question, and sterilisation as a means of solving the alcohol problem. My analysis builds on the common and recurring topics and themes in these conferences. Occasional comparisons with the pre-war conferences build on a previous study on the anti-alcohol conferences during the years 1885–1913.^4^ Previous research and theoretical aspects will be discussed with each theme.

**Table 1.** Conference Proceedings

Year	Title	City
1920	Proceedings of The Fifteenth International Congress against Alcoholism (CP 1920)	Washington, DC
1921	Compte-rendu du XVI congrès international contre l’alcoolisme (CP 1921)	Lausanne
1923	Compte-rendu du XVIIe congrès international contre l’alcoolisme (CP 1923)	Copenhagen
1925	Proceedings of the International Congress against Alcoholism (CP 1925)	Geneva
1928	Compte-rendu du XIXe congrès international contre l’alcoolisme (CP 1928)	Antwerp
1934	Proceedings of the Twentieth International Congress on Alcoholism (CP 1934)	London
1937	—	Warsaw
1939	Proceedings of the Twenty-Second International Congress against Alcoholism (CP 1939)	Helsinki

I will next briefly discuss the formulation of the alcohol problem and the transnational framework of the conferences. This will be followed by a discussion of the proposals to remedy the problem of alcohol. The last empirical section will historically contextualise the alcohol problem in relation to modernisation efforts, state-ideological currents and contemporary aspirations to explain the alcohol question in scientific terms. The article concludes with a round-up of the arguments.

## The Alcohol Problem and the Conferences

In comparison with the period around the turn of the century, the alcohol problem was rather played down as a political question in the interwar years.^5^ A great many countries introduced some form of retail control on alcohol in the course of the 1910s, and this trend was further boosted by the strive for national efficiency and sober warfare during the First World War.^6^ When several countries (Russia, Iceland, Norway, Finland and the United States) also introduced their own versions of prohibition during or soon after the war, the future looked bright for one of the key objectives of the radical temperance movement.^7^ This also contributed to a relative weakening of, or at the very least to a more passive role for, the temperance movement which had left such a clear mark on the anti-alcohol conferences of the first decades. As the political scientist Mark Lawrence Schrad has shown, the number of temperance organisations at these conferences already peaked at the 1911 meeting in The Hague, falling away remarkably toward the end of the period studied here.^8^

The conferences were important in several respects. They showed that the alcohol question had become such an important social political question that these large-scale meetings were warranted. The meetings had served as key sites for international knowledge and policy dissemination in the alcohol field ever since the very first conference in 1885 and continued to do so for another hundred years. They were the key and frequently held meetings for researchers, government officials and NGO representatives to gather around a topic which, since the late nineteenth century, had been described as a critical issue for Western societies.

Despite its weakened status, the temperance movement nevertheless had a clear role in the conferences. Representatives of various temperance organisations regularly appeared as speakers, and several conferences also discussed the movement as such.^9^ The spirit of the temperance movement continued to rest upon the conferences, which were *against* alcohol rather than non-partisan discussions *about* alcohol (the exception was the 1934 conference in London, which like the London conference of 1907 went under the heading of an International Congress *on* Alcoholism). The anti-alcohol line was made even clearer at the 1920 conference in Washington DC, which established that this was ‘distinctly a Congress *against* alcoholism’.^10^ The message was enhanced on many occasions, as several presentations dwelled on the harmful effects of alcohol on the individual and society. Alcohol was ‘a narcotic, a poison, and an abnormal drug’, with terrible effects: ‘it diminishes that moral, mental, and physical standard so necessary to the good of mankind’.^11^ Alcohol caused disease, death and criminality, damaged the offspring, led to mental illness, moral depravity and juvenile delinquency, and destroyed societies and civilisations.^12^

It mattered a great deal that the battle against alcohol was being wielded on an international forum. As Schrad argues, it helped to make the demands appear universal, as a fight between good and evil, rather than as a result of provincial interest formations.^13^ Alternatively, one can observe, as the historian Ian Tyrrell claims, how ‘[t]he impact of industrialization, commercial prosperity, and the growth of rapid communications sharpened intellectual interest in matters international and transnational’.^14^ The anti-alcohol movement was also just one of many international movements from the 1800s onwards that were organised around various social issues. The study of this transnational history has won increasing favour in recent historical research.^15^

The international arena, the transnational collaboration and the national sphere where actual policies were worked out were all part of the same picture. The national and the international were not at the opposing ends of the spectrum. Rather, internationalism demanded a certain nationalism: internationalism could be understood as collaboration *between* nations (as opposed to cosmopolitanism which had an indifferent relationship to the nation).^16^ Stefan-Ludwig Hoffman has identified this as a ‘nationalism backed by moral universalism’.^17^ And as the historian Robert Hohner has shown, such nationalism could appear as a kind of imperialism, which anchored matters close to American hearts in an international context and was happy to export the practice of prohibition to the rest of the world.^18^ US Secretary of the Navy Josephus Daniels summarised the American stance eloquently in the 1920 conference in Washington, DC:

Men of America are concerned for the welfare of men in Africa, and men in Asia, and men in Europe. And so this international body, powerless to legislate for any government, still has about it the power to educate, the power to inspire, and it has the purpose to say that what is evil to ignorant peoples and child races is evil to the educated people of the most advanced races.^19^

American enthusiasm about the international arena as an opportunity to mediate American experiences was understandably and especially palpable in the conference on home ground in 1920, when the long battle of the temperance movement had been crowned with nationwide prohibition. Finally, here was an opportunity for ‘the women of the world [to] strike hands with the men of the world, and they will march together until Prohibition shall be a fact in the constitution of every nation under the sun’.^20^

Historian Virginia Berridge argues that in Britain, too, research questions and discussions about control and legislation were similarly posed in an international and comparative context and that the anti-alcohol conferences were a natural arena for such debates.^21^ The interwar years are here labelled as a time of ‘continuing—and growing—internationalism of the alcohol question and of research’.^22^ Despite this, and unlike narcotics, international agreements did not regulate the alcohol traffic of the interwar years. The only exceptions were the various restrictions on the alcohol trade in and to the European colonies—just like before the First World War.^23^ The rhetoric of international solidarity was overflowing at the first post-war conference: ‘we are living in a time of international interest, we are learning that no nation can live to itself, that our problems are your problems and that your problems are our problems’.^24^ Such internationalism would never really reach the same enthusiastic heights, and it became increasingly clear in the 1930s that many conference participants represented nations on a collision course.

## Debating Solutions: From Moral Suasion to Sterilisation

The rhetoric of war was never far away, and the war demanded its dues: soldiers, workers and citizens had to stay sober. As Lloyd George, British Chancellor of the Exchequer had observed in 1915, ‘We are fighting Germany, Austria, and Drink; and as far as I can see the greatest of these three deadly foes is Drink’.^25^ Some pre-war problem formulations, such as the coupling of alcohol and national efficiency or motherhood, were also strengthened by the war.^26^ Conference delegates argued that the dire material circumstances had made people regard alcohol as an unnecessary luxury; that the war had helped to foster a sense of solidarity which was favourable to the temperance cause; or that people had simply come to realise during the more or less dry war years that they lived happier and better lives without alcohol.^27^ But alcohol was still used and misused, and one of the suggested solutions to this problem was moral suasion.

The moral ground of the temperance movement was more often than not similar to missionary religiosity. As the sociologists Pekka Sulkunen and Katariina Warpenius have put it, many ‘found it quite easy to amalgamate teetotalism into their own religious doctrines’.^28^ Abstinence and religiosity shared several points of contact, which is also evident in the conference delegates’ background organisations, including the Anti-Saloon League and Woman’s Christian Temperance Union (WCTU).^29^ It was also made clear that religion could become a useful resource in the temperance work: ‘Without religion there is no basis of authority above the human will, and without authority higher than the will of man there is no law and no morality’, claimed the Catholic prelate Regis Canevin, for example.^30^ The prohibition in the United States was interpreted as a manifestation of religious inspiration, and in the 1920 conference in Washington, DC Christian citizens were encouraged to involve themselves in society and politics to solve the problem of drunkenness.^31^

Similar to the pre-war conferences, temperance education—of the youth in particular and often as part of school work—came to have a central role in solving the alcohol question.^32^ But it is obvious that talk of temperance education was rather toned down in comparison with the earlier conferences.^33^ Maybe this was because the question offered few new angles of approach and it was therefore seen as well and truly ‘debated to death’, as the liberal politician and physician Curt Wallis had claimed at the 1907 conference in Stockholm.^34^

Young people and women were perfect representatives of the alcohol question, both as its tender victims and as its would-be solution. Women drank less than men, but they were nevertheless often the focus, sometimes as mothers, when the alcohol question was debated.^35^ As the Canadian Chief Justice of the Supreme Court argued in the 1920 conference, women were ‘the most directly affected, the most immediately exposed victim[s] of alcohol’.^36^ According to the American Democratic politician William Jennings Bryan, women would therefore drive the temperance question. Indeed, according to Bryan, American prohibition would have been introduced much earlier had it been decided by women.^37^ Female qualities and duties were regularly extolled as promoting temperance: women were exemplary builders of character and now that more countries had granted them political citizenship, they were able to drive these questions as voters and legislating politicians. Women’s responsibility for home and children were in the end the arguments that sealed their role in temperance work.^38^ In this context, home was not only a private family sphere but also ‘an ever widening circle’.^39^ Home and nation were part of a larger context in which women were tasked with nurturing both their own children and the characteristics of the population as a whole; women were ‘the guardians of the race’, ultimately responsible for any ‘influence upon race deterioration’.^40^

There were far fewer conference presentations on alcohol treatment in the interwar years than there had been before the First World War.^41^ This may be because remarkably few treatment institutions had survived the war, in the English-speaking world at least, and there were therefore fewer treatment units to provide treatment experiences. Social work researcher Jim Baumohl and sociologist Robin Room have analysed the impact of the war on this development and have shown that institutional treatment was unaffected in those countries which remained outside the war, such as Sweden and Switzerland. At the same time, one also needs to pay attention to what institutional treatment consisted of. Baumohl and Room quote the 1937 study by the physician Robert Fleming, according to whom responsibility for treatment rested with different authorities in different countries:

To epitomize, perhaps unfairly, in Sweden social workers, in Germany the geneticists, in Switzerland the moralists, and in Vienna an overworked psychiatrist do the actual work, while in England the community has a laisser-faire attitude and no one is responsible.^42^

Conference presentations on institutional treatment often came from countries such as Sweden and Finland, which had defined the alcohol problem in social terms and relied on social retraining. Some presentations also came from the Soviet Union, where it became possible in 1927 to commit alcohol misusers into coercive treatment on social grounds.^43^

When the United States joined the ranks of countries with prohibition, lines between legitimate and illegitimate alcohol consumption were redrawn. The enemy became more easily identified, as it now appeared in the shape of organised crime. This was also illustrated in the 1925 conference in Geneva, when a number of delegates discussed alcohol smuggling.^44^ Further, alcohol producers and their backers were seen as a threat to sobriety: it was the alcohol trade that was the main factor behind widespread alcoholism, claimed two French physicians in London in 1934.^45^ The breweries were powerful bodies, and their economic resources could manoeuvre both governments and the press.^46^ As the Methodist preacher Purley Baker aptly put it at one of the anti-alcohol conferences: ‘All great moral reforms that seriously affect the monetary interest of a large group of people quickly assume a political aspect …’.^47^ Had it not been for ‘the business judgment of man’, the United States would have introduced prohibition many years sooner.^48^ And in France, for example, it was almost impossible to ignore the many interests of the wine producers when the alcohol problem was being tackled. French alcohol consumption had reached record levels at the turn of the century, and the French temperance movement was weak and splintered.^49^ One conference presentation went as far as claiming that the efforts of the temperance movement went against the French mentality:

Finally, three traits of the French mentality constitute an added difficulty: the individualism of the Frenchman, which makes him hesitate to enroll himself under a banner; the instinct of independence, which often turns him away from signing an engagement of abstinence; and the fear of the ridiculous, which makes him afraid to affirm his anti-alcoholic convictions.^50^

Most participating countries had by this stage however introduced some kind of alcohol controls. In Britain, the war experiences helped to shift the focus from individual alcohol misuse to collective alcohol control.^51^ The conferences debated large-scale consumption studies as points of departure for various alcohol-political initiatives.^52^ Similarly, the conferences frequently got to hear accounts of the different national restrictive systems or prohibition.^53^

Still, conflicts between the various systems were obvious. By the interwar period, many in the Scandinavian temperance movement had finally accepted the Gothenburg system, which aimed to remove the profit interest from the alcohol trade by introducing a non-profit alcohol retail monopoly, and which had long been seen as a wet alternative to prohibition.^54^ At the same time, the Gothenburg system also allowed an institutionalisation of (regulated) alcohol consumption, which further distanced the drinking Europe from the dry United States. Such conflicting aims were also evident in the conferences.^55^ Toward the end of the period studied here, one Swedish delegate found himself defending the state monopoly against criticism which claimed that this was to be seen as an alcohol-favouring policy rather than as a compromise between dry and wet alcohol policies.^56^

The Gothenburg system had been frequently debated at pre-war conferences, but prohibition started to dominate the discussions once it had been introduced in several countries.^57^ The 1920 conference in Washington, DC was almost taken over by those singing the praises of prohibition. The Persian delegate was able to put the matter in some perspective by referring to the 300-year ban on alcohol, decreed by the prophet Muhammad, but otherwise the credits were innumerable.^58^

Ideas about the degenerating impact of alcohol had circulated at least since the eighteenth century, and the historian William Bynum in fact traces them back to antiquity. The Lamarckian theory of inheritance—that acquired characteristics could be passed on to offspring—was admittedly being questioned at the time.^59^ But as far as some conference delegates were concerned, it was still held to be true that the alcohol misuser ‘belongs to an inferior stock’, that his ‘tendency to drink is based on an inborn inferiority’, and that his alcohol consumption in turn led to inferior offspring.^60^ Women in particular should abstain from consuming alcohol because of its degenerating effects.^61^

In the conferences before the First World War, such thoughts had not led to any consensus about suitable measures. While some saw in them a heavy argument in favour of public sobriety, there were others who would rather have restricted the procreation of alcohol misusers. Persuasion and internment were debated as suitable means, but sterilisations were not advocated.^62^ In the interwar period, turn-of-the-century hygienic movements turned to a state-sanctioned agenda, which then appeared in many different guises depending on the ideological setting of the state. Comprehensive public health measures against sanitary problems and unhealthy environments shared the platform with measures of a more race-biological nature. As the historian Martin Pernick has shown, it is therefore not particularly helpful to make a distinction between interwar eugenics and public health measures.^63^ The primacy of the collective was a common feature in both, and to Arthur Newsholme, one of the leading public health experts in Britain, this was also absolutely central in the development of civilised societies: ‘Each advance in civilization implies increasing communal, supplementary to personal, control and limitation of acts which for the individual may be innocent, but which have been shown by protracted experiment and experience to be inimical to communal well-being …’.^64^

On the rhetorical level, then, the ambitions expressed here were not that different from those in Nazi Germany. The uniformed German physician Erich Bruns stated in the 1939 conference in Helsinki that the national socialist worldview underpinned all thinking and action in Germany—and that health was not an individual matter but concerned the entire nation.^65^

But proposals for practical measures during this time show a radicalisation of racial hygiene, which had served as a frame of thought in the temperance work ever since the late 1800s. The war boosted ideas about national efficiency in several fields, and in Britain, for example, this was linked with population quality and the racial hygiene of future generations. In France the concept of race featured prominently in wartime debates on alcohol use, when the fear of losing the war was linked to prospects of alcohol-related degeneration and troubles of rebuilding the country after the war.^66^

Racial hygiene was an international phenomenon and not the sole province of any single ideology.^67^ Liberals first raised interest in Sweden before racial hygiene was picked up by radical socialist and agrarian circles. In the United States, an early wave of sterilisations in the racial hygienic spirit has been linked with progressive forces, while similar measures in Britain met with resistance: the Labour Party rather regarded racial hygiene as anti-working class. Racial hygiene was a similarly complicated concept within nations, too. For example, in Britain it responded to a more conservative idea of mankind, that people had to find their natural place in society or that divisions in society reflected divisions of ability, while also demanding radical social reform. Michael Freeden also identifies several common points of departure in social reform movements, socialist circles and the racial hygiene movement in Britain.^68^ In Germany, eugenics was integral to Nazi ideology, legitimating sterilisation and murder, though the forced sterilisation of alcohol misusers predated Nazism. The Weimar government’s aim to find savings in costly institutional care led to expanding psychiatric non-institutional care, which served to justify sterilising outpatients and those who were considered beyond cure.^69^

But racial hygiene did not necessarily result in sterilisations. The goal of promoting good qualities (positive eugenics) and obstructing bad qualities (negative eugenics) could also appear as birth control advice. The way the causal relation worked was central: should poverty and social problems be explained by poor inheritance or should social problems and poverty be understood as concurrent and socially dependent forces? The former was the predominant notion during the interwar period, which explained the interest to limit the procreation of socially troublesome groups.^70^ This is where sterilisations—forced or otherwise—became one of the means in Germany: ‘chronic alcoholics’ were sterilised because their alcohol misuse was a manifestation of social or psychopathic damage.^71^

The conferences debated relatively frequently the possibility of restricting the procreation of alcohol misusers. In his presentation, the physician Charles T. Stockard maintained that rather than being the problem, alcohol was the solution when it came to this ‘class of weak mediocre people’: ‘Alcohol is one of the things that will tend to eliminate bad individuals, and inasmuch as from an economic standpoint they may not do much good or amount to much, why not use this means of eradicating them?’^72^

Stockard’s proposal of passive but purposeful killing had been discussed and dismissed in Vienna by the well-known race biologist Auguste Forel as an ineffectual method as early as 1901.^73^ In the 1920 conference, too, the proposal met with polite resistance from delegates who preferred to stop ‘these defectives’ from procreating by confining ‘them to farms where they would be treated kindly and mercifully, in a sort of colony where they would be prevented from reproducing’.^74^ Inheritance and environment were debated as competing and complementary factors during the entire interwar period when an individual’s behaviour was to be made sense of. The usual conclusion was that biological inheritance was the stronger of the two and that alcohol misusers therefore should be prevented from parenthood.^75^ As internment was deemed to be complicated, ineffectual and costly, and as a marriage ban was seen as similarly ineffective, sterilisations appeared to be the most promising means. Germany had in 1934 adopted a law enabling forced sterilisation in serious cases of alcoholism.^76^ But with ‘the growth of genetic knowledge and with the arousal of a “Racial Conscience” in the minds of our citizens’, hopes were also pinned on voluntary sterilisation, ‘not as a punishment or a deprivation, but as a privilege, and as a way out of a great difficulty, and as a means of harmonising conflicting individual and racial interests’.^77^ One of the few dissenting voices came from the Catholic church, which held that sterilised drinkers would suffer from much eroded sexual control, that sterilisation would be an encroachment on a person’s free will and—with the backing of a papal announcement—that such measures were against nature.^78^

## Modernity, State Ideology and Science

In comparison with pre-war conferences, the alcohol problem was debated in much more concrete terms in the interwar years.^79^ This was in all likelihood due to war experiences, the aim to achieve both military and civic efficiency.^80^ The Catholic church still regarded an individual’s moral stature as an unquestioned element of the solution, but many more delegates preferred to advocate for practical responses which were depicted as ethically neutral. As Charles T. Stockard claimed, one could not approach this question ‘from an ethical or humanitarian standpoint; we’ve got to consider it on a scientific basis’.^81^ This is where sterilisations appeared as a technical solution to a practical problem, and this approach permeated several aspects of the alcohol problem.

As before the First World War, the demands of the modern society helped to define the alcohol problem.^82^ The ever-expanding mechanisation and Taylorism left their mark on the working environment where sobriety and efficiency were demanded from the workers.^83^ Alcohol had had its place in working life—at times even sanctioned by the employer—and it was not without friction that people now adjusted to the demands of the new era. Employers, too, could find this development problematic, as lumberjacks, for example, who had stopped drinking became wealthier and therefore took longer holidays. In a somewhat curious contribution in the 1920 conference, a representative of the American forest industry put his trust in the churches: if these materially contented young men could be made to marry or if they could be rendered culturally more refined, one could hope to increase their requirements in life and thereby encourage them to work more. Still, unconditional condemnation of alcohol in working life was much more common, all in the name of increased efficiency and safety.^84^

Leisure was another dilemma, and all the more so with increasingly shorter working hours.^85^ Leisure pursuits could come in all kinds of unwanted guises, such as the much debated ‘dancing nuisance’, with concomitant drinking and immorality.^86^ To remedy this, one needed new leisure interests, such as sports. But doing sports did not necessarily mean staying sober, which is why a number of conference papers talked about the benefits of alcohol-free sports. Increased motoring also promoted the virtues of non-drink driving.^87^

Modernity demanded its dues but also met its critics. The destructive but logical function of alcohol in industrial society had been addressed since Friedrich Engels’ studies on the condition of the English working class and the nature of alcohol as an escapist comforter.^88^ At times the conferences would raise these perspectives again, but preferably with the caveat that increased welfare, improved working conditions, better education and training, and a more varied leisure time had by now decreased the need for alcohol as a social anaesthetic. With such ideological positioning of alcohol policies it was natural that the representative of Soviet state socialism in the 1934 conference knew how this question would be best solved: improve the general living conditions, give people a more varied leisure time, offer them better housing, create public employment and social services.^89^

The two world wars remodelled the very structures of the warring nations and in many cases also the make-up of nations which managed to stay outside the wars. Warfare, military preparedness, trade embargos and rationing meant that the modern states had to assume new duties. The politically and administratively revitalised states also addressed the alcohol problem with a new conviction during the interwar period. The aspirations were announced loud and clear when one of the leaders of the British temperance movement, Theodore Neild, lamented in the 1920 conference that the nation did not always have enough power to carry out its policies; what was needed in such cases was nothing less than ‘complete control’.^90^ Interwar developments also led to varying levels of state potency. No wonder that in Helsinki in 1939, only a month before the Second World War broke out, Robert Hercod (Executive Director of ITB and IBAA in 1907–1950) acknowledged that the constitutional basis was remarkably different in, for example, Germany and Britain. The dictatorships had certain undeniable advantages: ‘In the totalitarian countries where the State takes upon itself the education of youth outside the school and the home, a vigorous attitude against alcohol and the abstinence of the youth leaders is of extreme importance.’^91^ This was confirmed by the German delegates Hans Seidel and Ernst Bauer, who witnessed how the German notion of the state had changed, thereby also changing the nature of involvement among the official bodies whose duty it was to fight alcohol misuse. This fight was now more comprehensive and was understood as the sum of all measures—‘die Gesamtsumme aller Massnahmen’—by the authorities who were to guide the people and the nation.^92^

The clearest indications of views on state control were seen when the conferences debated the liberal dilemma of collective rule for the benefit of the individual. This was not a major problem in the authoritarian states of the interwar period, but was also not a matter of principle for the democracies, either, when it came to dissecting their undemocratic ways of governing the colonial populations.^93^ The colonial powers could without problems make decisions on sales restrictions and prohibition; indeed, the question about alcohol consumption among ‘the Native Races’ was ‘a subject which *par excellence* is suitable for discussion at an International Conference’.^94^ At the same time, this care for the natives spoke of one’s own problems and the shortcomings of national politics. After all, ‘what is evil to ignorant peoples and child races is evil to the educated people of the most advanced races’.^95^

The alcohol problem was understood and solved in a national—and on many occasions in a nationalistic—context. The First World War fed on and reproduced nationalistic frames of understanding which survived the peace and continued to organise political thinking during the interwar period.^96^ In the conferences, this mindset could argue that alcohol use was a foreign custom or that solutions to the alcohol question were intended to preserve the national community, ‘die Volksgemeinschaft’.^97^ During the politically turbulent interwar period the nationalistic take on the alcohol question went hand in hand with increasingly obvious political positioning, especially in the 1930s when Nazi Germany was seeking to sell its ideological framing by the side of liberal and social democratic democracies and the communist Soviet Union. The reading of the conference proceedings, therefore, gives us a rather different picture of the interwar development, which Berridge has described as a period of growing internationalism.^98^ One obvious change during this period is in fact the decline of internationalism and the onslaught of nationalistic propaganda manoeuvres. The less important role of the transnational temperance movement and the rise of totalitarian regimes most likely contributed to this.

The totalitarian regimes also used the conferences as propaganda platforms, as when the Soviet ambassador to Britain, Ivan Mikhailovich Maisky, presented a paper in the 1934 conference in London on how his country had tackled the alcohol problem during the last decade:

This has been brought about by the development of the national economy, the successful fulfilment of the Five-Year Plan, the tremendous growth in education, the wiping out of unemployment and the collectivisation of the countryside. The health services, the prophylactic treatment of social ills, which has grown so tremendously in the last few years, the improvement in workers’ dwellings, the extensive house-building, the system of public feeding, which now embraces 20 million of the population, maternal and child welfare have all had their part in the decline of alcoholism.^99^

Also significant for this positive development were ‘the shock-work system, socialist competition, study, physical culture and sport, the mass of social events, living and working in a collective, the sense of participation in the work of the new social order’.^100^ Summing up, Maisky argued that this would solve the problem once and for all: ‘in the near future there is no doubt the problem of alcoholism will cease to exist altogether’.^101^ A commentary to the ambassador’s presentation also made it known that it was impossible to understand how the alcohol problem in the Soviet Union was being tackled unless one also understood the communist ideology which permeated the whole society.^102^

In Helsinki in 1939, the German delegates repeated and even upstaged the Soviet marketing coup. Several German presentations talked about the Nazi regime as enabling a holistic grip on the alcohol question.^103^ The German lawyer, race hygienist and co-author of the German Sterilisation Law of 1933, Falk Ruttke, painted a picture of a fateful development which had finally come to its end. The race hygienic theme which had been frequently debated in the previous conferences was now placed in a unique context when Ruttke purposely promoted the national socialist concern over one people within one nation: the *German* people. But the purpose was also—and here one can hear war knocking at the door—to guarantee space or ‘Lebensraum’ for this people.^104^ Out of this overarching goal, one could extract German policies against the misuse of alcohol and tobacco, which in many respects differed little from those measures which had long been debated in the anti-alcohol conferences. But they also promoted education about how alcohol and tobacco threatened to destroy the German race, giving this frequently repeated argument of degeneration a Nazi German appearance. The youth should therefore be made to realise that a healthy lifestyle was a national duty.

It was most of all the unquestioned role of the state that the German delegates emphasised. The state constituted a certain way of life or ‘Lebensform’ which permeated the German people’s lives and the very shape of the civilisation.^105^ Erich Bruns argued that the national socialist ideology which lay at the ground of all thinking and activity—‘alles Denkens und Handelns’—redefined the entire relationship between individual and collective. Health was no longer an individual concern but a concern of the nation. Also, physicians were no longer only intended for those who were ill but would now function as administrators of public health, as ‘Gesundheitsführer’.^106^

The Nazi German and the Soviet communist notions were explained in explicitly ideological terms, but the problem was constructed as an individual’s conflict with the collective in many countries. Problems conceived in such terms guarantee ideological solutions which will partly debate the legitimacy of the morally binding collective and partly anchor the solutions in this very legitimacy. And it is here that the scientific argument often stood the debate in good stead.

In this context, science and ideology should not be treated as opposing entities. I do not agree with Schrad’s view that the conferences represented either transnational advocacy networks or epistemic communities, the former described as ‘primarily [motivated] by shared *normative* understandings’ and the latter as sharing ‘scientific and *cognitive* understandings’.^107^ This distinction helps Schrad to establish that epistemic communities are only loosely connected to the political process, while transnational advocacy networks ‘actively seek social and political change based primarily on shared understandings of right versus wrong and good versus evil’.^108^ But, as previous research has shown, a scientific rendering of a question can also contribute to a de-ideologisation of sorts which enables a stronger political mobilisation in various questions.^109^ The interwar anti-alcohol conferences did indeed feature fewer presentations which conceived the alcohol problem in religious or general moral terms. Still, science was not necessarily able to determine whether something was true or not, and as the philosopher Roger Smith has shown, the biological sciences of the nineteenth and the twentieth centuries have been both descriptive and normative. Smith also points out that biologists’ references to ‘the body politic’ or ‘the social organism’ have sought to anchor ideologically preferable ideals in accounts of the natural order.^110^

As a frame of understanding and as a basis of state action, racial hygiene obviously depended on this scientific legitimacy. Historian of ideas Gunnar Broberg and historian Mattias Tydén have even claimed that racial hygiene made such inroads into contemporary societies because it expressed a more widespread reliance on science. And historian Yvonne Hirdman argues that scientific competence was a prerequisite in interfering with people’s lives during the 1930s whether they wanted it or not.^111^ Then as now, the positivist ideal of science strengthened time- and culture-bound notions on the society and the individuals, endowing them with traces of eternal truths. Or, as the historian Martin S. Pernick has described this in the context of the American movement of racial hygiene: ‘This widely shared faith in objectivity did not succeed in eliminating subjective values from medicine, but it did serve to delegitimise the openly political and ethical debate that is necessary if a culture is to assess its value judgments intelligently’.^112^

The anti-alcohol conferences valued the scientific argument highly. Conference delegates toned down their emotional aversion to alcohol, and when the repeal of American prohibition was being debated, it was made clear that the decision could not be based on ‘a wave of sentiment’ but that the matter should rest on ‘sound evidence’.^113^ This also applied to temperance education: to be successful, such education should abstain from ‘moral exhortation against drinking’ in favour of ‘scientific facts’.^114^ And when one discussed suitable methods of alcoholism treatment, the debate was able to draw on ‘modern scientific conception’.^115^ The temperance movement had already before the First World War complemented its ideological pathos with arguments of a scientific nature, continuing these efforts by, for example, collecting scientific facts through the Scientific Temperance Federation.^116^ Such methodical truth-seeking work may have lacked a certain fervour, but it was no longer possible to sell ‘facts which can not be thoroughly backed up by the best scientific evidence’.^117^

The passion for science reappeared when a somewhat misguided debate was said to have ‘wandered very far afield from the scientific discussion of the problem of alcoholism’.^118^ Science had also shown that the modern life demanded sobriety.^119^ Sterilising alcohol misusers was not an issue, either, that could be discussed ‘from an ethical or humanitarian standpoint’; it could only be seen ‘on a scientific basis’.^120^ Also, a shift from medical aspects to social factors was motivated by ‘a more scientific outlook on social phenomena in general’.^121^

With science on one’s side, it was possible to promote certain questions with much more credibility: ‘Science has taught us that even a moderate use of intoxicating liquor is harmful’, said William Jennings Bryan, thereby also settling the question of whether it was preferable to abstain from alcohol altogether or drink in moderation.^122^ But the fact that it was Bryan who flew the flag of science suggests caution. Bryan was a sworn enemy of Darwinism and the evolutionary theory, and played a significant role in the 1925 Scopes Trial on the right to teach evolutionary theory in American state-funded schools. Bryan did not dismiss science but rather argued that evolutionary theory was unscientific.^123^ But he also pleaded the authority of religion: ‘If you have any faith in the Bible, turn back through its pages and find that wine has been a mocker throughout the years.’^124^ Later at the same conference, Bryan admitted that he had always been ‘interested in the demonstrations of science’ but that he nevertheless felt that ‘the instinct is often more speedy in its action and more sure in its results than mathematical calculations’.^125^ His more traditional stance on the temperance question was backed by P. J. O’Callaghan, a representative of the Catholic Total Abstinence Union: ‘There is nothing real or great in the world but ideals; the rest of the world dies’.^126^ Or, as it was expressed in the 1934 conference in London: ‘Science and Idealism must go hand in hand’.^127^

There was thus no shared point of departure and strategy built on scientific grounds. It was not even certain that the conference delegates were speaking about the same question. As William White has shown, there was tremendous breadth in the conceptual understanding of the alcohol problem. Turn-of-the-century attempts to describe alcohol misuse as a disease had made few converts, and the problem formulation was also relatively disparate in the interwar years.^128^ As shown in a recent project on ‘addiction concepts’ in four European countries during the late nineteenth and early twentieth centuries, there was no conceptual common comprehension of ‘addiction’ between the countries—even if you limit this to the medical field.^129^ Not surprisingly then, this was also the case at the investigated conferences with their broader scope of perspectives.

The 1920 conference discussed alcohol’s ‘specific action on the human brain’ but alcoholism was still defined as, for example, ‘men’s abuse of alcohol’.^130^ In the last conference of the interwar period, one fascinating presentation offered the theoretical possibility that alcohol misuse could be seen as a particular manifestation of an overwhelming ‘Süchtigkeit’, an addiction which could also appear as a wandering urge (‘Wandertrieb’), kleptomania (‘Stehlsucht’), pyromania (‘Brandstiftungssucht’), a compulsive urge to commit murder (‘Mordsucht’), a dancing urge (‘Tanzsucht’) and as a general urge to seek pleasure (‘Vergnügungssucht’).^131^ People who might be seen as problem gamblers today were also included, as were those athletes and adventurers who yearned for a thrill.^132^ However, at the same conference—and in order to underline the breadth of contemporary understandings—it was also argued (typically enough by a Nordic representative) that it was extremely hard medically to determine alcoholism and that the problem should rather be approached from a social angle.^133^

## Conclusion

The First World War tested the internationalistic ambitions of popular movements. Much has been written about the disappointment within the labour movement when workers were sent to fight other workers in the bloodiest war known to contemporary mankind.^134^ The first post-war conferences nevertheless enjoyed an optimistic atmosphere, and American prohibition had obviously given the temperance movement something to be hopeful about. But when the 1920s turned to the 1930s, it became increasingly evident that the conferences were being transformed into arenas for various national agendas. Such shared problems as criminality, population characteristics, core functions of the state, and industrial efficiency were increasingly given a national stamp. And when the enthusiasm of the early years over the desirability for prohibition had waned, it was possible to see how the common endeavour to solve a common problem through a common agenda was exchanged for national solutions and outright propaganda pieces, where one’s own responses were marketed as a logical outcome of state ideological aspirations.

This was a transition period for the temperance movement. On the one hand, it was driven by a relatively vague religious yet still idealistic power. On the other hand, for both strategic and epistemological reasons it was influenced by scientific thought. The division was apparent across the whole field, from inspired Presbyterian ministers and Nazi philosophers of a mysterious bent to science-prone Baptists and sociologically-oriented communists. The radical responses of the racial hygienists made room for ideological convictions of varying colours, but they also shared a common faith in ethically neutral science. Sterilisations were propagated and practised as a reaction to individuals’ alcohol consumption in such democratic countries as Sweden, the United States and Weimar Germany. The national socialist takeover in Germany extended the eugenic arsenal to include murder.

The responses to the alcohol problem debated in the interwar conferences built on a combination of scientifically masked ideological conviction and ideologically inspired passion for science. The apparently neutral ethics that we can detect in such thinking was manifested in its most extreme forms in the horrors of the Second World War. The more brutal varieties of race biology were discredited in the racist genocide committed by the Nazis, but the ‘scientification’ of the alcohol problem had only been hinted at during the interwar period. While the rise of the influential alcoholism movement after the Second World War was equally grounded in social, economic and ideological currents, it was often portrayed as a triumph of science. Now that various means of collective alcohol restrictions had been found wanting, the alcohol problem needed new solutions. After the Second World War, the relationship would yet again be shaken between the alcohol misusing individual and a new kind of society.

## Footnotes

^1^George Snow, ‘Temperance in Imperial Russia’, in Jack S. Blocker Jr., David M. Fahey and Ian R. Tyrrell, eds, *Alcohol and Temperance in Modern History. An International Encyclopedia. Vol. 2* (Santa Barbara: ABC-CLIO, 2003), 529–32.

^2^Harry G. Levine, ‘The Alcohol Problem in America: From Temperance to Alcoholism’, *British Journal of Addiction*, 1984, 79, 109–19; Nils Christie and Kettil Bruun, *Den gode fiende. Narkotikapolitikk i Norden* (Oslo: Univ.-forl, 1985); Henrik Tham, ‘Narkotikakontroll som nationellt projekt’, *Nordisk Alkoholtidskrift*, 1992, 9, 86–97; Johan Edman, ‘The Ideological Drug Problem’, *Drugs and Alcohol Today*, 2013, 13, 9–19.

^3^All conference volumes barring one have been accessible at The Swedish Council for Information on Alcohol and Other Drugs (CAN). Despite all efforts, it has not been possible to trace the report of the 1937 conference in Warsaw.

^4^Johan Edman, ‘Temperance and Modernity: Alcohol Consumption as a Collective Problem 1885–1913’, *Journal of Social History*, 2015, 49, 20–52.

^5^See, for example: John Greenaway, *Drink and British Politics since 1830. A Study in Policy Making* (Basingstoke: Palgrave Macmillan, 2003).

^6^Mark Lawrence Schrad, *The Political Power of Bad Ideas. Networks, Institutions, and the Global Prohibition Wave* (Oxford: Oxford University Press, 2010).

^7^Mark Lawrence Schrad, *Vodka Polictics. Alcohol, Autocracy, and the Secret History of the Russian State* (New York: Oxford University Press, 2014); Helgi Gunnlaugsson and John F. Galliher, ‘Prohibition of Beer in Iceland: An International Test of Symbolic Politics’, *Law & Society Review*, 1986, 20, 335–54; Per Ole Johansen, ‘The Norwegian Alcohol Prohibition—a Failure’, *Journal of Scandinavian Studies in Criminology and Crime Prevention*, 2013, 14, 46–63; John H. Wuorinen, ‘Finland’s Prohibition Experiment’, *Annals of the American Academy of Political and Social Science*, 1932, 163, 216–26; Ian Tyrrell, ‘The US Prohibition Experiment: Myths, History and Implications’, *Addiction*, 1997, 92, 1405–9.

^8^Mark Lawrence Schrad, *The Prohibition Option. Transnational Temperance and National Policymaking in Russia, Sweden and the United States* (University of Wisconsin–Madison, 2007).

^9^See, for example: E. C. Hennigar, ‘Peculiar Colours in the Japanese Movement’, *CP [Conference Proceedings] 1934*.

^10^‘Discussion’, *CP 1920*, 321.

^11^De Lancy Carter, ‘Beer and Light Wines as Intoxicants’, *CP 1920*, 53.

^12^August Ley, ‘Alcohol and Criminality’, *CP 1920*; Paul Maurice Legrain, ‘Nervous and Mental Diseases in Their Relations to Alcoholism’, *CP 1920*; Carlos Gibson, ‘Response’, *CP 1920*; M. F. Boulenger, ‘Délinquance juvénile et alcoolisme familial’, *CP 1921*; Dr Vervæck, ‘L’alcoolisme et la criminalité immorale chez l’adulte’, *CP 1921*; Mme Gerken-Leitgebel, ‘La jeunesse et la moralité sexuelle’, *CP 1921*; Dr Deets-Pickett, ‘La moralité sexuelle aux Etats-Unis depuis la prohibition’, *CP 1921*; Harald Westergaard, ‘La statistique et l’influence de l’alcoolisme sur la santé’, *CP 1923*; Taav. Laitinen and Agnes Bluhm, ‘Etudes experimentales relatives à l’influence de l’alcool sur la postérité’, *CP 1923*; Taav. Laitinen, ‘Expériences sur l’alcool et l’hérédité’, *CP 1928*; J. Sôrra, ‘Influence de l’alcoolisme des parents sur le nombre des naissances, la croissance et le développement des descendants’, *CP 1928*; Fr. Grant, ‘L’excitabilité de l’écore cérébrale chez les descendants d’alcooliques’, *CP 1928*; M. Kask, ‘Action de l’alcool sur les animaux et ses relations avec l’hypo- et l’hyperfonction des glandes endocrines’, *CP 1928*; Louise McIleoy, ‘Alcohol and Womanhood’, *CP 1934*; R. Bandel, ‘Alkoholverbrauch und spezifische Männersblickheit’, *CP 1934*.

^13^Schrad, *The Political Power of Bad Ideas*.

^14^Ian Tyrrell, *Reforming the World. The Creation of America’s Moral Empire* (Princeton, NJ: Princeton University Press, 2010), 19.

^15^For an introduction to the study of transnational history, see: Margaret E. Keck and Kathryn Sikkink, *Activists Beyond Borders. Advocacy Networks in International Politics* (Ithaca, NY: Cornell University Press, 1998); Akira Iriye, ‘Transnational History’, *Contemporary European History*, 2004, 13, 211–22; Akira Iriye, *Global and Transnational History. The Past, Present, and Future* (Basingstoke: Palgrave Macmillan, 2013).

^16^Michael Billig, *Banal Nationalism* (London: Sage, 1995); Tom Nairn, *Faces of Nationalism. Janus Revisited* (London: Verso, 1997); Samuel Edquist, *Nyktra svenskar. Godtemplarrörelsen och den nationella identiteten* (Uppsala: Acta Universitatis Upsaliensis, 2001).

^17^Stefan-Ludwig Hoffman, ‘Nationalism and the Quest for Moral Universalism. German Freemasonry, 1860–1914’, in Martin H. Geyer and Johannes Paulmann, eds, *The Mechanics of Internationalism. Culture, Society, and Politics from the 1840s to the First World War* (London: German Historical Institute, 2001), 259–84, 282.

^18^Robert A. Hohner, *Prohibition and Politics. The Life of Bishop James Cannon, Jr.* (Columbia: University of South Carolina Press, 1999). See also: Ian Tyrrell, ‘Prohibition, American Cultural Expansion, and the New Hegemony in the 1920s: An Interpretation’, *Social History*, 1994, 27, 413–45.

^19^Josephus Daniels, ‘Address’, *CP 1920*, 133.

^20^Deborah Livingston, ‘Response’, *CP 1920*, 408.

^21^Virginia Berridge, ‘The Impact of War 1914–1918’, *British Journal of Addiction*, 1990, 85, 1017–22.

^22^Virginia Berridge, ‘The Inter-War Years: A Period of Decline’, *British Journal of Addiction*, 1990, 85, 1023–35, 1025.

^23^Kettil Bruun, Lyn Pan and Ingemar Rexed, *The Gentlemen’s Club. International Control of Drugs and Alcohol* (Chicago: University of Chicago Press, 1975); Liat Kozma, ‘The League of Nations and the Debate over Cannabis Prohibition’, *History Compass*, 2011, 9, 61–70; James H. Mills, ‘Cocaine and the British Empire: The Drug and the Diplomats at the Hague Opium Conference, 1911–12’, *The Journal of Imperial and Commonwealth History*, 2014, 42, 400–19; Philippe Bourmaud, ‘Les faux-semblants d’une politique internationale: la Société des Nations et la lutte contre l’alcoolisme dans les mandats (1919–1930)’, *Canadian Bulletin of Medical History*, 2013, 30, 69–90.

^24^Cora Frances Stoddard, ‘Response’, *CP 1920*, 415.

^25^‘Editorial’, *British Journal of Nursing*, 1915, 54, 269.

^26^Catherine J. Kudlick, ‘Fighting the Internal and External Enemies: Alcoholism in World War I France’, *Contemporary Drug Problems*, 1985, 12, 129–58; Schrad, *The Political Power of Bad Ideas*; Patricia E. Prestwich, *Drink and the Politics of Social Reform. Antialcoholism in France since 1870* (Palo Alto: Society for the Promotion of Science and Scholarship, 1988); Berridge, ‘The Impact of War 1914–1918’.

^27^Agnes E. Slack, ‘The Women of Europe against Alcoholism’, *CP 1920*; Francois Lemieux, ‘Response’, *CP 1920*; Mr Larsen-Ledet, ‘Address’, *CP 1920*; H. Carter, ‘Effets hygiéniques et sociaux des mesures prises contre l’alcool pendant la guerre. En Grande-Bretagne’, *CP 1921*; Dr Vogel, ‘Effets hygiéniques et sociaux des mesures prises contre l’alcool pendant la guerre. En Allemagne’, *CP 1921*; Dr Hindhede, ‘Effets hygiéniques et sociaux des mesures prises contre l’alcool pendant la guerre. Au Danemark’, *CP 1921*.

^28^Pekka Sulkunen and Katariina Warpenius, ‘Reforming the Self and the Other: The Temperance Movement and the Duality of Modern Subjectivity’, *Critical Public Health*, 2000, 10, 423–38, 427.

^29^Margot Opdycke Lamme, ‘Alcoholic Dogs and Glory for All: The Anti-Saloon League and Public Relations, 1913’, *Social History of Alcohol and Drugs*, 2007, 21, 138–59; Ian Tyrrell, ‘Women and Temperance in International Perspective: The World’s WCTU, 1880s–1920s’, in Susanna Barrows and Robin Room, eds, *Drinking. Behavior and Belief in Modern History* (Berkeley: University of California Press, 1991), 217–40.

^30^Regis Canevin, ‘The Churches of America in the Movement against Alcoholism: The Roman Catholic Church’, *CP 1920*, 137.

^31^William F. Anderson, ‘The Churches of America in the Movement against Alcoholism: The Protestant Churches’, *CP 1920*; John G. Woolley, ‘Address’, *CP 1920*.

^32^See, for example: Prof. Zmrhal, ‘Temperance Instruction in European Schools’, *CP 1920*; M. Wightman, ‘La préparation à l’enseignement antialcoolique’, *CP 1921*; F. Goebel, ‘Comment amener la jeunesse à s’abstenir d’alcool par les efforts des jeunes gens eux-mêmes’, *CP 1923*; Fedor Mikic, ‘Comment intéresser les adolescents à la lutte contre l’alcoolisme?’, *CP 1928*; F. Rudolf, ‘Wie kann die Wirkung unserer Aufklärungsarbeit vertieft werden?’, *CP 1934*; Iwan Bolin, ‘Grundsätze des alkoholgegnerischen Unterrichts’, *CP 1939*.

^33^Edman, ‘Temperance and Modernity’.

^34^Curt Wallis, ‘Skolan och alkoholfrågan’, *CP 1907*, 49.

^35^Prestwich, *Drink and the Politics of Social Reform*; David W. Gutzke, ‘Gender, Class, and Public Drinking in Britain during the First World War’, *Social History*, 1994, 27, 367–91; Berridge, ‘The Impact of War 1914–1918’; Kudlick, ‘Fighting the Internal and External Enemies’.

^36^Lemieux, ‘Response’, 358.

^37^W. J. Bryan, ‘Response’, *CP 1920*.

^38^Slack, ‘The Women of Europe against Alcoholism’; Anna A. Gordon, ‘Woman’s Relation to the Antialcohol Movement in America’, *CP 1920*; Mme la Princesse Puzyna, ‘La femme au parlement et la lutte contre l’alcoolisme’, *CP 1923*; Laura Pearson, ‘The Role of the Woman-Citizen in the Fight against Alcoholism’, *CP 1923*; Agnes Slack, ‘Discussion’, *CP 1923*; Milda Kempels, ‘Wie kann man die Frauen für den Kampf gegen den Alkoholismus gewinnen’, *CP 1939*. See also four presentations under the heading ‘Women’s Work for Temperance’ in *CP 1934*.

^39^Pearson, ‘The Role of the Woman-Citizen in the Fight against Alcoholism’, 165.

^40^McIlroy, ‘Alcohol and Womanhood’, 92.

^41^Edman, ‘Temperance and modernity’.

^42^Robert Fleming, ‘The Management of Chronic Alcoholism in England, Scandinavia and Central Europe’, *New England Journal of Medicine*, 1937, 216, 279–89. Quoted in: Jim Baumohl and Robin Room, ‘Inebriety, Doctors, and the State: Alcoholism Treatment Institutions before 1940’, *Recent Developments in Alcoholism*, 1987, 5, 135–74, 165.

^43^See, for example: Harry Venoja, ‘Principles of Legislation on the Treatment of Inebriates’, *CP 1939*; Olof Kinberg, ‘The Treatment of Inebriates in Sweden’, *CP 1939*; S. S. Salmensaari, ‘The Treatment of Inebriates in Finland’, *CP 1939*; Ivan Mikhailovich Maisky, ‘Alcoholism in the USSR and the Measures Designed to Combat It’, *CP 1934*.

^44^No less than 14 papers discussed this, see\ *CP 1925*, 65–128.

^45^Dr Legrain and Dr Paul Dauphin, ‘L’activité internationale du trafic de l’alcool’, *CP 1934*.

^46^A. Holitscher, ‘Beer as a Cause of Alcoholism’, *CP 1920*; W. J. Bryan, ‘Discussion’, *CP 1920*.

^47^Purley A. Baker, ‘Political Aspects of the Prohibition Movement in America: Partizan and Non-Partizan Effort’, *CP 1920*, 74.

^48^Bryan, ‘Response’, 402.

^49^Legrain, ‘Nervous and Mental Diseases in their Relations to Alcoholism’; Prestwich, *Drink and the Politics of Social Reform*; Michael R. Marrus, ‘Social Drinking in the Belle Epoque’, *Journal of Social History*, 1974, 7, 115–41.

^50^Jean Méteil, ‘The Antialcoholic Struggle in France’, *CP 1920*, 164.

^51^Berridge, ‘The Impact of War 1914–1918’. However, as criminologist Henry Yeomans has argued, British alcohol regulation appears rather weak in comparison with regulations in other countries at the time and considering the pressures of war: Henry Yeomans, ‘Providentialism, the Pledge and Victorian Hangovers’, *Law, Crime, and History*, 2011, 1, 95–107.

^52^Tapio Voionmaa, ‘An International Survey on the Production and Consumption of Alcoholic Beverages’, *CP 1939*.

^53^See, for example: Robert Hercod, ‘Prohibition in Russia’, *CP 1920*; Einar H. Kvaran, ‘La prohibition de l’alcool en Islande’, *CP 1921*; K. Aro, ‘La prohibition de l’alcool en Finlande’, *CP 1923*; Dr Dahlgren, ‘Le système du contrôle individuel de la vente de l’eau-de-vie en Suède’, *CP 1928*; Haven Emerson, ‘Les résultats de la prohibition américaine au point de vue hygiénique’, *CP 1928*.

^54^Robin Room, ‘The Formulation of State Alcohol Monopolies and Controls: Case Studies in Five Nations’, *Contemporary Drug Problems*, 1985, 12, 1–9.

^55^R. Hercod, ‘Conflicts Between Liquor Exporting States and States which have Taken Prohibitive or Severely Restrictive Measures against the Liquor Traffic’, *CP 1925*; Hohner, *Prohibition and Politics*.

^56^Erik Englund, ‘Disinterested Management’, *CP 1939*; H. Cecil Heath, ‘Disinterested Management’, *CP 1939*.

^57^Edman, ‘Temperance and Modernity’.

^58^Abdul Ali Khan, ‘Responses to Addresses of Welcome’, *CP 1920*.

^59^W. F. Bynum, ‘Alcoholism and Degeneration in 19th Century European Medicine and Psychiatry’, *British Journal of Addiction*, 1984, 79, 59–70; Karl Mann, Derek Hermann and Andreas Heinz, ‘One Hundred Years of Alcoholism: The Twentieth Century’, *Alcohol & Alcoholism*, 2000, 35, 10–15; Martin S. Pernick, ‘Eugenics and Public Health in American History’, *American Journal of Public Health*, 1997, 87, 1767–72.

^60^H. Gachot, ‘Alcoolisme et eugenisme’, *CP 1934*, 212.

^61^McIleoy, ‘Alcohol and Womanhood’, 92.

^62^Edman, ‘Temperance and Modernity’.

^63^Pernick, ‘Eugenics and Public Health in American History’. On public health as an ever-widening concept and an agenda, see also: Martin Gorsky, ‘Public Health in Interwar England and Wales: Did it Fail?’, *Dynamis*, 2008, 28, 175–98.

^64^Arthur Newsholme, ‘The Place of the Alcohol Question in Social Hygiene’, *CP 1928*, 20.

^65^Erich Bruns, ‘Ärzte und Alkohol’, *CP 1939*.

^66^Berridge, ‘The Impact of War 1914–1918’; Berridge, ‘The Inter-War Years’; Kudlick, ‘Fighting the Internal and External Enemies’; Prestwich, *Drink and the Politics of Social Reform*.

^67^Robert Proctor, *Racial Hygiene. Medicine under the Nazis* (Cambridge, MA: Harvard University Press, 1988).

^68^Gunnar Broberg and Mattias Tydén, *Oönskade i folkhemmet. Rashygien och sterilisering i Sverige* (Stockholm: Gidlund, 1990); Mark A. Largent, *Breeding Contempt. The History of Coerced Sterilizations in the United States* (New Brunswick: Rutgers University Press, 2008); John Macnicol, ‘Eugenics and the Campaign for Voluntary Sterilization in Britain Between the Wars’, *Social History of Medicine*, 1989, 2, 147–69; Roger Smith, ‘Biology and Values in Interwar Britain: C. S. Sherrington, Julian Huxley and the Vision of Progress’, *Past & Present*, 2003, 178, 210–42; Michael Freeden, ‘Eugenics and Progressive Thought: A Study in Ideological Affinity’, *The Historical Journal*, 1979, 22, 645–71.

^69^Mann, Hermann and Heinz, ‘One Hundred Years of Alcoholism’; Michael Burleigh, ‘Psychiatry, German Society, and the Nazi “Euthanasia” Programme’, *Social History of Medicine*, 1994, 7, 213–28.

^70^Julie Grier, ‘Eugenics and Birth Control: Contraceptive Provision in North Wales, 1918–1939’, *Social History of Medicine*, 1998, 11, 443–58; Greta Jones, ‘Eugenics and Social Policy between the Wars’, *The Historical Journal*, 1982, 25, 717–28.

^71^Burleigh, ‘Psychiatry, German Society, and the Nazi “Euthanasia” Programme’, 219.

^72^Charles T. Stockard, ‘Latest Scientific Investigation in America of the Action of Alcohol’, *CP 1920*, 375.

^73^Auguste Forel, ‘Die Alkoholfrage als Cultur- und Rassenproblem’, *CP 1901*.

^74^C. W. Saleeby, ‘Discussion’, *CP 1920*, 378.

^75^G. H. Carpenter, ‘Alcohol, Heredity and Environment’, *CP 1934*; Gachot, ‘Alcoolisme et eugenisme’.

^76^Erich Schröder, ‘Neue rassenhygienische Wege zur Bekämpfung des Alkoholismus in Deutschland’, *CP 1934*; Falk Ruttke, ‘Erbpflege und Bekämpfung von Alkoholschäden im grossdeutschen Reich’, *CP 1939*.

^77^J. Bond, ‘Discussion’, *CP 1934*, 224.

^78^Rev. Hays, ‘Alcoholism and a Healthy Future Generation’, *CP 1934*.

^79^Edman, ‘Temperance and Modernity’.

^80^Newsholme, ‘The Place of the Alcohol Question in Social Hygiene’; F. A. Brandt, ‘Alcohol and Railways’, *CP 1923*.

^81^Stockard, ‘Latest Scientific Investigation in America of the Action of Alcohol’, 375.

^82^David T. Courtwright and Timothy A. Hickman, ‘Modernity and Anti-Modernity: Drug Policy and Political Culture in the United States and Europe in the Nineteenth and Twentieth Centuries’, in Geoffrey Hunt, Maitena Milhet and Henri Bergernon, eds, *Drugs and Culture. Knowledge, Consumption and Policy* (Burlington: Ashgate, 2011), 213–24. See, for example: Eugene Lyman Fisk, ‘The Relationship of Alcohol to Modern Health Ideals’, *CP 1920*.

^83^Berridge, ‘The Impact of War 1914–1918’; Berridge, ‘The Inter-War Years’; Kudlick, ‘Fighting the Internal and External Enemies’; Tyrrell, ‘Prohibition, American Cultural Expansion, and the New Hegemony in the 1920s’; Sarah W. Tracy and Caroline Jean Acker, ‘Introduction: Psychoactive Drugs—an American Way of Life’, in Sarah W. Tracy and Caroline Jean Acker, eds, *Altering American Consciousness. The History of Alcohol and Drug Use in the United States, 1800–2000* (Amherst: University of Massachusetts Press, 2004), 1–30.

^84^William S. Bennet, ‘Response’, *CP 1920*; Newsholme, ‘The Place of the Alcohol Question in Social Hygiene’; Courtenay C. Weeks, ‘Alcohol in Relation to Professional and Industrial Efficiency’, *CP 1939*.

^85^van der Meulen, ‘The Organisation of the Leisure Time of the Labourer and its Significance for the Struggle against Alcohol’, *CP 1939*.

^86^Edwin Wide, ‘International Sports Champions and the Use of Alcohol’, *CP 1939*, 220.

^87^*Ibid*.; A. Mallwitz, ‘Influence des boissons alcooliques sur le travail sportif’, *CP 1921*; F. Riémain, ‘Le sport dans la lutte contre l’alcoolisme’, *CP 1921*; Marcel Bergeron, ‘L’alcool et les sports’, *CP 1928*; R. Cove Smith, ‘Alcohol and Sport’, *CP 1934*; Newsholme, ‘The Place of the Alcohol Question in Social Hygiene’; H. M. Vernon, ‘The Influence of Alcohol on Motor Driving’, *CP 1934*; Courtenay C. Weeks, ‘Alcohol and Motor Transport’, *CP 1934*.

^88^Wolfgang Schivelbusch, *Paradiset, smaken och förnuftet. Njutningsmedlens historia* (Stockholm: Alba, 1982).

^89^van der Meulen, ‘The Organisation of the Leisure Time of the Labourer and its Significance for the Struggle against Alcohol’; John F. Kramer, ‘Enforcement of Prohibition in the United States’, *CP 1920*; Newsholme, ‘The Place of the Alcohol Question in Social Hygiene’; Maisky, ‘Alcoholism in the USSR and the Measures Designed to Combat It’.

^90^Theodore Neild, ‘The Alcohol Problem in Great Britain during and after the War’, *CP 1920*, 157.

^91^R. Hercod, ‘Forms of State Participation in the Fight against Alcoholism’, *CP 1939*, p. 56. A more critical view on the authoritarian states from the same conference is to be found in: van der Meulen, ‘The Organisation of the Leisure Time of the Labourer and its Significance for the Struggle against Alcohol’.

^92^Hans Seidel and Ernst Bauer, ‘Die Formen der Beteiligung des Staates am Kampf gegen den Alkoholismus. Deutschland’, *CP 1939*, 58.

^93^Bourmaud, ‘Les faux-semblants d’une politique internationale’.

^94^Charles F. Harford, ‘Alcoholism and the Native Races as an International Problem’, *CP 1920*, 291. A few examples of many papers discussing this: Henry W. Peabody, ‘The Protection of Native Races from Alcoholism’, *CP 1920*; Mr Blackburn and M Julien, ‘Faits récents touchant le traffic de l’alcool aux colonies’, *CP 1923*; A. E. Blackburn, ‘Some Facts and Figures Concerning the Liquor Traffic in the British Colonies and Protectorates of Africa’, *CP 1925*; K. McLay, ‘Alcohol and the Native Races. The Maoris of New Zealand’, in: *CP 1934*.

^95^Daniels, ‘Address’, 133.

^96^Arno Mayer, ‘Post-War Nationalisms 1918–1919’, *Past & Present*, 1966, 34, 114–26.

^97^W. P. Wei, ‘The Movement against Alcoholism in China’, *CP 1920*; Dr Paulstich and Dr Ernst Gabriel, ‘Das Problem der Süchtigkeit’, *CP 1939*, 37.

^98^Berridge, ‘The Inter-War Years’.

^99^Maisky, ‘Alcoholism in the USSR and the Measures Designed to Combat It’, 287.

^100^*Ibid*., 288.

^101^*Ibid*.

^102^Thomas Murray, ‘Notes on the Report on Alcoholism in the USSR, and the Measures Designed to Combat It’, *CP 1934*.

^103^Seidel and Bauer, ‘Die Formen der Beteiligung des Staates am Kampf gegen den Alkoholismus. Deutschland’.

^104^Ruttke, ‘Erbpflege und Bekämpfung von Alkoholschäden im grossdeutschen Reich’, 121.

^105^*Ibid*., 128.

^106^Erich Bruns, ‘Ärzte und Alkohol’, *CP 1939*, 171.

^107^Schrad, *The Political Power of Bad Ideas*, 33. On transnational advocacy networks and epistemic communities, see also: Keck and Sikkink, *Activists Beyond Borders*; Peter M. Haas, ‘Introduction: Epistemic Communities and International Policy Coordination’, *International Organization*, 1992, 46, 1–35.

^108^Schrad, *The Political Power of Bad Ideas*, 33.

^109^Robert Martin, ‘Context and Contradiction: Toward a Political Theory of Conceptual Change’, *Political Research Quarterly*, 1997, 50, 413–36; Michael Th. Greven, ‘Dimensions of Politics. A Critique of the Common One-Dimensional Concepts of Politics’, *Redescriptions*, 2001, 5, 89–112; Johan Edman, ‘What’s in a Name? Alcohol and Drug Treatment and the Politics of Confusion’, *Nordic Studies on Alcohol and Drugs*, 2009, 26, 339–53; Edman, ‘The Ideological Drug Problem’.

^110^Smith, ‘Biology and Values in Interwar Britain’, 220.

^111^Broberg and Tydén, *Oönskade i folkhemmet*; Yvonne Hirdman, *Att lägga livet tillrätta. Studier i svensk folkhemspolitik* (Stockholm: Carlssons, 1990).

^112^Pernick, ‘Eugenics and Public Health in American History’, 1770.

^113^Fisk, ‘The Relationship of Alcohol to Modern Health Ideals’, 39. See also: J. Scharffenberg, ‘L’organisation des recherches scientifiques sur la question de l’alcool’, *CP 1921*.

^114^Cora Frances Stoddard, ‘Scientific Temperance Instruction in the Public Schools of the United States’, *CP 1920*, 312.

^115^Venoja, ‘Principles of Legislation on the Treatment of Inebriates’.

^116^Edman, ‘Temperance and Modernity’.

^117^Cora Frances Stoddard, ‘Response’, *CP 1920*, 459.

^118^Charles A. Rosewater, ‘Discussion’, *CP 1920*, 64.

^119^Newsholme, ‘The Place of the Alcohol Question in Social Hygiene’.

^120^Stockard, ‘Latest Scientific Investigation in America of the Action of Alcohol’, 375.

^121^Kinberg, ‘The Treatment of Inebriates in Sweden’, 307.

^122^William Jennings Bryan, ‘Address’, *CP 1920*, 348.

^123^Edward J. Larson, *Summer for the Gods. The Scopes Trial and America’s Continuing Debate over Science and Religion* (Cambridge, MA: Harvard University Press, 1998).

^124^Bryan, ‘Address’, 354.

^125^Bryan, ‘Response’, 402.

^126^P. J. O’Callaghan, ‘Response’, *CP 1920*, 460.

^127^‘A New Departure at the Congresses’, *CP 1934*, 103.

^128^William White, ‘The Lessons of Language. Historical Perspectives on the Rhetoric of Addiction’, in Tracy and Acker, eds, *Altering American Consciousness*, 33–60; Tracy and Acker, ‘Introduction’; William White, ‘The Rebirth of the Disease Concept of Alcoholism in the 20th Century’, *Counselor*, 2000, 1, 62–6.

^129^Virginia Berridge *et al.*, ‘Addiction in Europe, 1860s–1960s: Concepts and Responses in Italy, Poland, Austria, and the United Kingdom’, *Contemporary Drug Problems*, 2014, 4, 551–66. The more elaborated studies on each nation can be found in the special issue on concepts of addiction in Europe, 1860s–1930s: *Social History of Alcohol and Drugs*, 2014, 28. See also the recently published thematic issue on the historical conceptual framing of drug and alcohol problems in the Nordic countries: *Nordic Studies on Alcohol and Drugs*, 2014, 31.

^130^Ley, ‘Alcohol and Criminality’, 105; Canevin, ‘The Churches of America in the Movement against Alcoholism, 137.

^131^Paulstich and Gabriel, ‘Das Problem der Süchtigkeit’, 33 f.

^132^The historian David T. Courtwright maintains that the governing ideas about alcohol, tobacco and other drugs (ATOD) at this time emphasized their differences rather than commonalities. The conference papers examined here are a clear exception to this tendency. David T. Courtwright, ‘Mr. ATOD’s Wild Ride: What Do Alcohol, Tobacco, and Other Drugs have in Common?’, *Social History of Alcohol and Drugs*, 2005, 20, 105–40.

^133^Venoja, ‘Principles of Legislation on the Treatment of Inebriates’.

^134^Susan Milner, *The Dilemmas of Internationalism. French Syndicalism and the International Labour Movement, 1900–1914* (New York: Berg, 1990). As Eric Hobsbawn has shown, the picture is nevertheless far from uncomplicated. The disappointment among the leaders of the labour movement was not necessarily shared by the workers or socialists as a whole. Also, the anti-war sentiments in some socialist circles testified rather of pacifism than peace-oriented internationalism. Eric J. Hobsbawm, ‘Working-Class Internationalism’, in Frits van Holthoon and Marcel vad der Linden, eds, *Internationalism in the Labour Movement 1830–1940. Volume I* (Leiden: Brill 1988), 3–16.

